# Linking FANTOM5 CAGE peaks to annotations with CAGEscan

**DOI:** 10.1038/sdata.2017.147

**Published:** 2017-10-03

**Authors:** Nicolas Bertin, Mickaël Mendez, Akira Hasegawa, Marina Lizio, Imad Abugessaisa, Jessica Severin, Mizuho Sakai-Ohno, Timo Lassmann, Takeya Kasukawa, Hideya Kawaji, Yoshihide Hayashizaki, Alistair R. R. Forrest, Piero Carninci, Charles Plessy

**Affiliations:** 1 RIKEN Center for Life Science Technologies, Division of Genomics Technologies, Yokohama 230-0045, Japan; 2 RIKEN Omics Science Center, Yokohama 230-0045, Japan; 3 RIKEN Preventive Medicine and Diagnosis Innovation Program, Wako 351-0198, Japan

**Keywords:** Transcriptomics, Bioinformatics

## Abstract

The FANTOM5 expression atlas is a quantitative measurement of the activity of nearly 200,000 promoter regions across nearly 2,000 different human primary cells, tissue types and cell lines. Generation of this atlas was made possible by the use of CAGE, an experimental approach to localise transcription start sites at single-nucleotide resolution by sequencing the 5′ ends of capped RNAs after their conversion to cDNAs. While 50% of CAGE-defined promoter regions could be confidently associated to adjacent transcriptional units, nearly 100,000 promoter regions remained gene-orphan. To address this, we used the CAGEscan method, in which random-primed 5′-cDNAs are paired-end sequenced. Pairs starting in the same region are assembled in transcript models called CAGEscan clusters. Here, we present the production and quality control of CAGEscan libraries from 56 FANTOM5 RNA sources, which enhances the FANTOM5 expression atlas by providing experimental evidence associating core promoter regions with their cognate transcripts.

## Background & Summary

CAGE (Cap Analysis Gene Expression^1^) is the method of choice for studying gene regulation through quantitative analysis of transcription start sites (TSS, sequence ontology term 0000315)^2^. By sequencing the 5′ end of cDNA-converted capped RNAs, CAGE enables the identification of core promoter regions and 5′ end transcriptional activity. Large scale application of CAGE by the FANTOM consortium to nearly 2,000 human RNA sources including primary cells, whole-tissue extracts and cell lines^3,4^ identified nearly 200,000 core promoter regions active within the human genome^5^.

Although CAGE enables the location of TSS at a single nucleotide resolution, the determination of their connection to downstream known gene structures or to independent novel RNAs is limited to positional computational inference and low-throughput gene-by-gene experimental validations. Half (101,893/201,802) of the FANTOM5’s active core promoter regions did not co-localise within a reasonable distance with 5′ termini of annotated gene models. To experimentally associate these orphan core promoter regions to transcriptional units, we employed *CAGEscan*^6^, an approach in which paired-end sequencing of the 5′ end of cDNA-converted capped RNAs with their cognate randomly priming sites enables the unequivocal association of individual TSS to transcripts exons. In a previous project, focused on analysing the translatome of Purkinje neurons in rat^7^, the CAGEscan approach annotated 43 % of the core promoters active in rat’s Purkinje neurons that we detected but had no by direct overlap with Ensembl transcripts.

**Figure 1:**
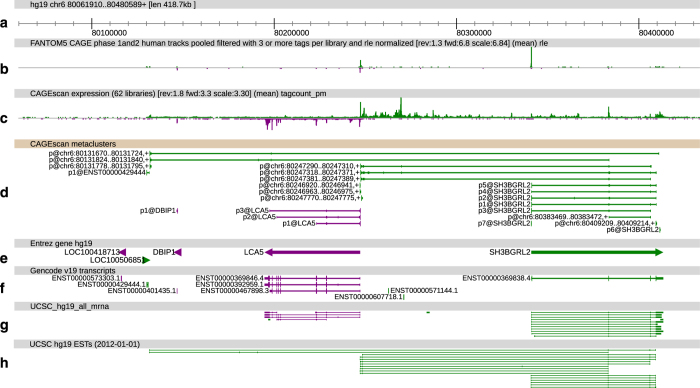
ZENBU view of CAGEscan data. CAGEscan clusters revealing new promoters for the SH3BGRL2 gene. Features on the plus and minus strand are displayed in green and purple respectively. Promoter regions of interest are highlighted with ellipses in track D. (**a**) Genomic coordinates. (**b**) FANTOM5 CAGE signal as a quantitative histogram. (**c**) CAGEscan CAGE signal. (**d**) CAGEscan meta-clusters, combining pairs for all libraries. The name of the seed CAGE peak is indicated on the left of each cluster. (**e**) NCBI Gene bodies. (**f**) GENCODE 19 annotations. (**g**) GenBank mRNA sequences. (**h**) EST sequences supporting the CAGEscan clusters.

Here, we selected 56 RNA sources which upon FANTOM5 CAGE profiling revealed the greatest levels of transcriptome diversity and prepared individual CAGEscan libraries, with 6 of these 56 RNA sources prepared in duplicate (see Table 1). Using the FANTOM5 core promoter atlas as seed, we clustered the CAGEscan paired-end reads in a collection of 112,315 models called *CAGEscan clusters*, by collating all the pairs whose alignment started in the same FANTOM5 CAGE peak. To de-orphanise FANTOM5 promoters, we intersected the CAGEscan clusters with GENCODE 18 gene models. Of the 85 % that intersected, 33,632 clusters had no annotation in FANTOM5, thus revealing novel and alternative promoters to known genes. We made these data available along with the FANTOM5 CAGE atlas data, as well as ready for manual inspection and analysis via the ZENBU genome browser http://fantom.gsc.riken.jp/zenbu/gLyphs/#config=ZkJi4RdBAFhnsudxePrZxD (see Fig. 1).

## Methods

**Figure 2:**
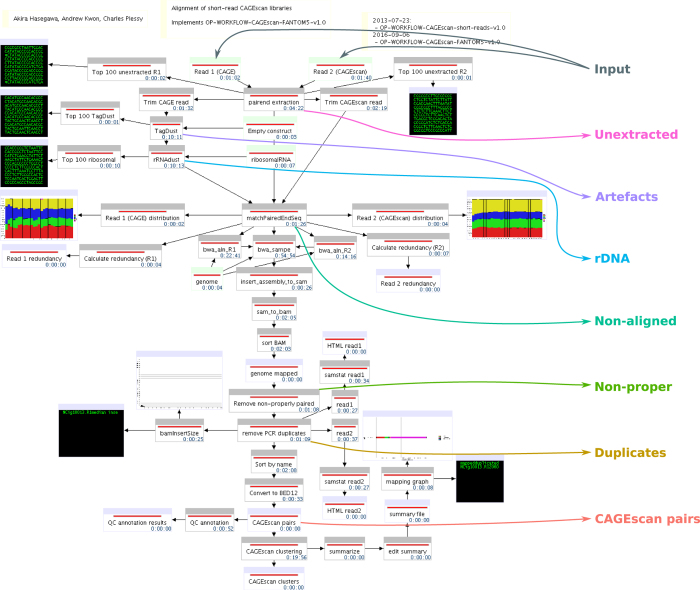
FANTOM5 CAGEscan processing workflow. Processing pipeline. The diagram made of boxes connected by black arrows displays the MOIRAI workflow completed for one (NCig10013) of the 62 CAGEscan libraries. The coloured text and arrows overlayed on the diagram represents the points where the main alignment statistics are calculated to summarise the number of read pairs passing all the filters (CAGEscan pairs) or discarded at each step of the processing pipeline (Unextracted, rDNA, Artefacts, Non-aligned, Non-proper, Duplicates).

All human samples used in the project were either exempted material (available in public collections or commercially available), or provided under informed consent. All non-exempt material is covered under RIKEN Yokohama Ethics applications (H17–34 and H21-14). The CAGEscan libraries were prepared as described earlier^8^. In brief, 500 ng of RNA were reverse-transcribed in presence of random primers and template-switching oligonucleotides, amplified by PCR and sequenced paired-end (2×36 nt) on Illumina GAIIx sequencers, one sample per lane. The barcode sequence GCTATA, present in every sample, acted as the spacer that we introduced in ref. 9 to decrease the amount of strand-invasion artefacts. The paired-end sequences were then processed with the MOIRAI workflow system^10^, with a template implementing the workflow OP-WORKFLOW-CAGEscan-FANTOM5-v1.0, described below and in Fig. 2.

For each pair, the first (CAGE) and second (CAGEscan) reads in FASTQ format were demultiplexed. The first 9 bases of the CAGE reads were trimmed as they contain the sample barcode and the template-switching linker. CAGEscan paired-end reads that did not contain the exact barcode and linker sequences were discarded. The first 6 bases of the CAGEscan reads were trimmed, because they originate from the random primers and not the cDNAs, and therefore are prone to errors caused by mismatches during the hybridisation to the RNAs, that are well tolerated by the reverse-transcriptase^11^.

The CAGE and CAGEscan reads were then filtered independently with the TagDust program version 1.13 (ref. 12), using the sequences of empty constructs and primers as artefact library. They were then compared to reference sequences of ribosomal genes (GenBank: U13369.1) using the rRNAdust program version 1.03. Reads whose mates were discarded by these two filters were then removed.

FASTQ formatted cleaned paired-end reads were then aligned on the human genome version hg19 with BWA version 0.7.15 (ref. 13) using standard parameters, except that the maximum insert length (−a) was set to 2 Mbp to allow pairs to map on different exons, and that insert size detection was disabled (−A). Extra header records (for SQ: AS and for RG: CN, ID, LB, PU, SM, and PL) were added to ease processing and tracking. The resulting BWA SAM formatted alignments were then converted to BAM format, and unmapped as well as non-properly paired CAGE reads were discarded (flag 0×42). The resulting ‘CAGEscan pairs’ provide individual experimental information on the association of a single-nucleotide-resolution TSS with the body of a gene product.

The CAGEscan pairs were then converted to BED12 format using the program pairedBamToBed12 version 1.2, in which the score field is the sum of the mapping qualities of each read of the pair. They were then assembled into CAGEscan clusters using the CAGEscan-Clustering script version 1.2 and the Phase 1+2 FANTOM5 DPI CAGE peaks as seeds. The CAGEscan-Clustering script also takes advantage of the BED12 format, reporting the number of CAGEscan paired-end reads used to assemble each cluster via the score field and the name and position of the seeding CAGE peak via the name, thickStart and thickEnd fields respectively. Finally, the CAGEscan clusters from all libraries were then combined into a single global assembly of ‘meta-clusters’ using the same program and output in BED12 files where the score indicates the number of libraries contributing data to each meta-cluster.

### Code availability

The MOIRAI workflow template used to process the libraries is available as a supplemental XML file (Data Citation 1). MOIRAI enabled the design of a complete data processing pipeline based on the following softwares: FASTX-Toolkit (http://hannonlab.cshl.edu/fastx_toolkit/), TagDust 1.13 (ref. 12), rRNAdust 1.03 (http://fantom.gsc.riken.jp/5/sstar/Protocols:rRNAdust) (note that for new projects, we recommend TagDust 2 instead of TagDust 1 and rRNAdust), BWA 0.7.15-r1140^13^, SAMtools 0.1.19-44428cd^14^, pairedBAMtoBED12 1.2 (https://github.com/Population-Transcriptomics/pairedBamToBed12, Data Citation 2), CAGEscan-Clustering.pl 1.2 (https://github.com/nicolas-bertin/CAGEscan-Clustering, Data Citation 3) and promexinstats.sh for the annotation (see Data Citation 1). The software above and standard Unix tools are sufficient to re-implement the pipeline in a different workflow system.

## Data Records

Each CAGEscan library is described with a Sample and Data Relationship Format (SDRF) record, together with the rest of the FANTOM5 data^15^. For each library, raw sequences in FASTQ format, alignment data in BAM format (including unmapped reads), CAGEscan pairs in BED12 format, CAGEscan clusters in BED12 format and alignment statistics in plain text tabulation-delimited triples (subject, predicate, object), are available in the FANTOM5 data repository (http://fantom.gsc.riken.jp/5/datafiles/phase2.3/basic/). The raw sequences have also been deposited to DDBJ Sequence Read Archive (Data Citation 4).

## Technical Validation

**Figure 3:**
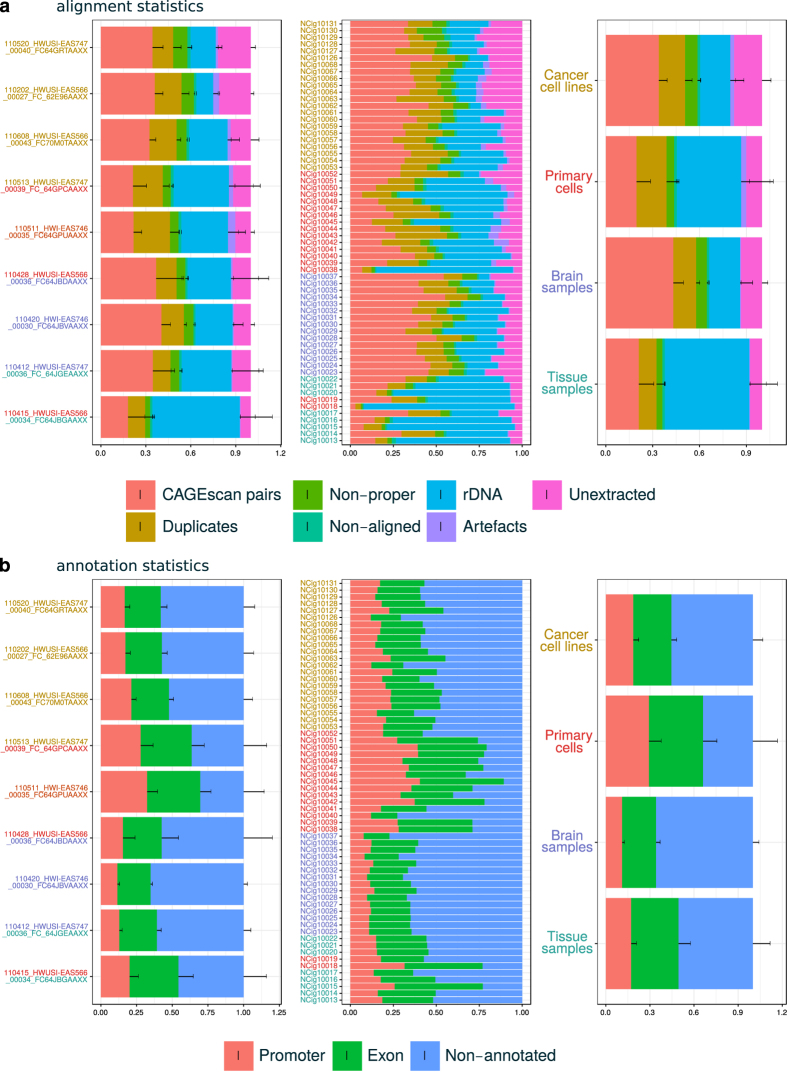
Alignment and annotation statistics. Quality control statistics. (**a**) Fraction of pairs passing all filters (CAGEscan pairs) or discarded at key steps of the processing pipeline (see Fig. 2). The central block of stack bars represents each library individually. The left block aggregates them by sequencing batch, named by the sequencing run identifier. The right block aggregates the libraries by sample type. Each sample type is represented by one colour, that is also used to colour the library identifiers and the sequence identifiers in the other blocks. Batches comprising multiple types are indicated by multiple colours. (**b**) Fraction of pairs starting in a Promoter, Exon, or Other (non-promoter, non-exon) region.

We derived individual library alignment statistics from the MOIRAI data processing pipeline (see Table 1 and Figs 2 and 3a). The statistics count the number of reads discarded at key steps of the processing. ‘Unextracted’ are pairs where the linker was not found, ‘Artefacts’ are pairs that matched the artefact library, or had a low complexity, ‘rDNA’ are pairs that matched the reference rDNA locus (including rRNAs and their spacer regions), ‘Non-aligned’ are pairs where one or both mates were not aligned to the genome, and ‘Non-proper’ are pairs where the mates were not aligned in head-to-head orientation within 2 Mbp. ‘Duplicates’ are the pairs removed during the deduplication step. That is, when there are *n* pairs with identical coordinates, 1 is kept and *n*−1 are discarded as ‘Duplicates’. These statistics show that the amount of PCR duplicates was not larger than the number of CAGEscan pairs, suggesting that the libraries prepared in this study have not been fully exhausted by sequencing.

The library alignment statistics, as well as statistics describing the distribution of CAGEscan TSSs on GENCODE 19 annotations (Fig. 3b), also suggest that the biological nature of the samples (cancer cell lines, primary cells, tissue samples and brain tissue) strongly influenced the performance of the CAGEscan protocol used in this study. Albeit displaying the best performance in terms of alignment (largest fraction of CAGEscan pairs), brain tissue derived samples had the lowest rate of known promoters overlapping start sites, hinting at a much greater diversity of alternative promoters usage in human brain. However, since, in this study, all brain tissue derived samples were taken from a single donor, this observation may result from technical batch effect rather than being a general feature of the nature of human brain transcriptome.

**Figure 4:**
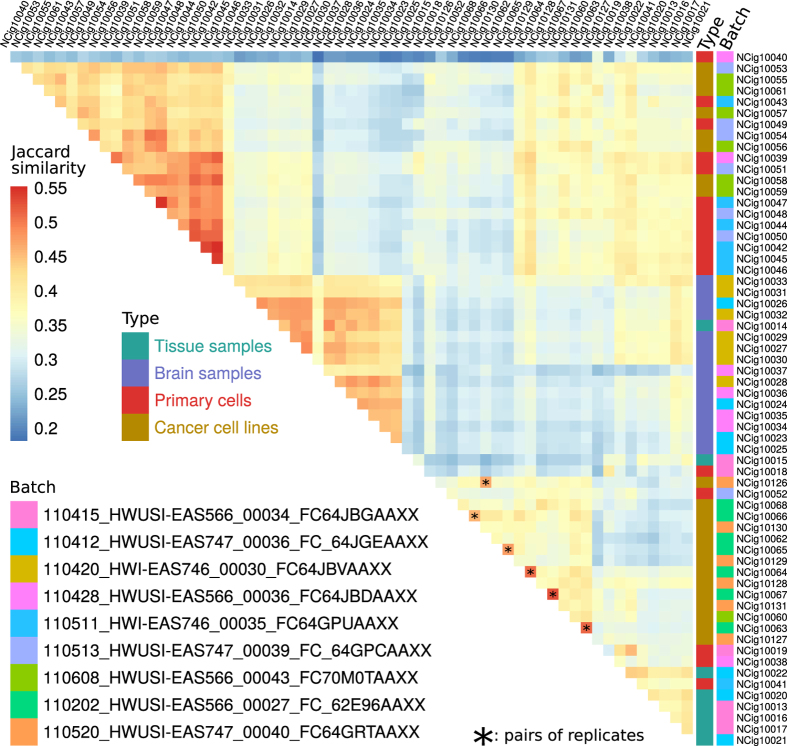
Similarity between libraries. Heatmap of the Jaccard similarity indexes computed between each pair of libraries. Sample type and batches are indicated by a colour code near library names, and pairs of replicates are indicated by an asterisk superimposed to the square displaying their similarity index.

To assess the reproducibility and consistency of our libraries, we computed a Jaccard similarity index between the lists of FANTOM5 CAGE peaks detected in each possible pair of libraries. For each sample analysed in duplicate, the library with the highest similarity was the replicate (Fig. 4). Hierarchical clustering of the libraries tended to group the samples by type rather than by batch. Accordingly, library NCig10014, typed as ‘Tissue’ together with other samples obtained from Ambion’s FirstChoice Human Total RNA Survey Panel, and containing its brain RNA pool, clustered with the donor-derived ‘Brain samples’. Together with the similarity of replicates, this provides confidence that the data reflects the biological contents of the libraries and not batch effects.

## Usage Notes

We have seeded the CAGEscan clustering with FANTOM5 CAGE-defined core promoter regions, however alternative seeding strategies could be envisioned. The 5′ ends of the CAGEscan pairs themselves could be clustered by peak calling and used as a seed, which is the default mode of operation of the pairedBamToBed12 tool. Foregoing the discovery of alternative promoters, CAGEscan clusters could also be seeded using promoter regions defined by GENCODE models. To discover potential enhancer-associated non-coding RNAs, region corresponding to FANTOM5 enhancers^16^ could also be used.

We used a simple alignment strategy that did not take splicing into account. Thus, pairs overlapping splice junctions could not be mapped and CAGEscan clusters lack coverage at the beginning and end of each exon, but this only mildly impacts the main purpose of the method. In addition, since the CAGEscan pairs are anchored at the 5′ end of the transcripts, splice junctions occurring close to the TSS may render some whole loci unmappable. Indeed, transcripts databases such as GENCODE reveal splice junctions very near to the TSS. Trimming the CAGE reads to 20 nt rescued some loci, but other loci were lost due to the decrease of alignment stringency (data not shown).

One of the most striking differences between the HeliScopeCAGE-based FANTOM5 CAGE data and the nanoCAGE-based FANTOM5 CAGEscan data is a larger amount of start sites in the gene body, far from the promoter. This can be explained by the lower stringency of the nanoCAGE protocol, which uses template-switching for capturing 5′ ends from limiting amounts of samples^6^, where the HeliScopeCAGE protocol, that uses CAP Trapper^17^, would not be possible. Readers curious about the position of the random priming site, indicated by the end position of the CAGEscan pairs, will notice that their distribution is very far from random. Control experiments performed using different batches of random primers ordered by different makers confirmed that the quality of the oligonucleotides was not in question (data not shown). In the latest version of the nanoCAGE protocol^18^, this problem was solved by the fragmentation of the cDNAs by the ‘tagmentation’ method. Altogether, we recommend to use our latest protocol for making new libraries.

In this study, the CAGEscan libraries were prepared using the nanoCAGE method, but the CAGEscan workflow, which can use any paired-end sequencing of CAGE libraries were the 3′ sequencing read is at a random position in the cDNA, can be applied to other publicly available dataset, for instance made with the RAMPAGE method^19^.

## Additional Information

**How to cite this article:** Bertin, N. *et al.* Linking FANTOM5 CAGE peaks to annotations with CAGEscan. *Sci. Data* 4:170147 doi: 10.1038/sdata.2017.147 (2017).

**Publisher’s note:** Springer Nature remains neutral with regard to jurisdictional claims in published maps and institutional affiliations.

## Supplementary Material



## Figures and Tables

**Table 1:** Summary of the libraries prepared.

	**Source Name**	**Description**	**Total**	**Unextracted**	**Artefacts**	**rDNA**	**Non aligned**	**Non proper**	**Duplicates**	**Promoter**	**Exon**	**Non annotated**
NCig10013	10002-101A5	SABiosciences XpressRef Human Universal Total RNA, pool1	12,980,474	865,232	53,578	8,620,490	303,381	336,125	914,464	352,035	557,012	978,157
NCig10014	10012-101C3	brain, adult, pool1	10,041,908	789,460	56,053	3,579,617	156,712	499,657	1,967,826	479,841	1,007,678	1,505,064
NCig10015	10016-101C7	heart, adult, pool1	17,071,911	657,065	67,493	12,680,315	189,587	360,818	1,791,722	341,589	679,820	303,502
NCig10016	10026-101D8	testis, adult, pool1	12,778,881	735,402	56,663	8,828,467	229,250	357,108	761,921	321,059	575,393	913,618
NCig10017	10030-101E3	retina, adult, pool1	7,438,898	983,120	49,209	2,016,040	91,931	396,226	1,395,594	341,442	574,800	1,590,536
NCig10018	11210-116A4	Smooth Muscle Cells—Aortic, donor0	16,069,580	636,805	76,008	14,079,255	126,255	163,413	505,347	152,188	219,216	111,093
NCig10019	12176-128I7	Whole blood (ribopure), donor090325, donation1	11,721,271	745,633	59,630	5,626,776	168,819	557,490	1,749,239	502,772	701,914	1,608,998
NCig10020	10019-101D1	lung, adult, pool1	13,194,146	853,743	90,406	8,943,567	141,998	347,985	819,745	309,787	599,126	1,087,789
NCig10021	10022-101D4	prostate, adult, pool1	11,134,114	746,116	64,413	6,095,769	167,516	486,536	1,152,553	366,192	716,651	1,338,368
NCig10022	10025-101D7	spleen, adult, pool1	8,339,981	852,309	62,976	3,130,575	143,638	431,102	1,038,248	402,237	785,577	1,493,319
NCig10023	10150-102I6	medial frontal gyrus, adult, donor10252	4,512,027	960,285	25,800	467,916	81,388	389,764	534,385	224,651	506,845	1,320,993
NCig10024	10151-102I7	amygdala, adult, donor10252	6,314,079	858,652	33,503	1,112,179	112,044	450,492	801,134	321,988	702,445	1,921,642
NCig10025	10153-102I9	hippocampus, adult, donor10252	5,068,313	892,716	32,233	861,081	80,184	360,888	643,485	241,503	530,452	1,425,771
NCig10026	10154-103A1	thalamus, adult, donor10252	8,151,958	817,751	35,872	2,262,386	95,945	505,911	1,296,220	377,511	716,479	2,043,883
NCig10027	10155-103A2	medulla oblongata, adult, donor10252	9,999,787	1,397,247	78,367	2,366,286	132,873	610,522	1,541,331	446,644	906,737	2,519,780
NCig10028	10157-103A4	parietal lobe, adult, donor10252	9,456,143	936,084	71,291	1,471,269	153,886	694,447	1,387,954	463,355	1,096,767	3,181,090
NCig10029	10158-103A5	substantia nigra, adult, donor10252	7,656,663	1,078,146	59,251	2,360,698	85,562	425,712	1,188,939	344,322	602,764	1,511,269
NCig10030	10159-103A6	spinal cord, adult, donor10252	9,651,183	888,981	79,200	2,801,018	116,324	594,991	1,353,570	442,320	903,764	2,471,015
NCig10031	10160-103A7	pineal gland, adult, donor10252	7,577,434	1,011,960	65,055	1,343,792	103,948	545,004	944,389	348,323	744,959	2,470,004
NCig10032	10161-103A8	globus pallidus, adult, donor10252	11,489,499	821,077	80,387	4,015,936	130,251	673,588	1,632,249	469,685	916,587	2,749,739
NCig10033	10162-103A9	pituitary gland, adult, donor10252	8,630,256	970,964	49,755	1,932,932	124,341	563,707	1,591,606	455,105	847,858	2,093,988
NCig10034	10163-103B1	occipital cortex, adult, donor10252	9,407,509	905,254	44,694	1,193,623	136,650	708,731	1,223,230	432,869	1,030,595	3,731,863
NCig10035	10164-103B2	caudate nucleus, adult, donor10252	6,816,957	1,102,408	38,186	869,711	109,813	476,042	1,310,808	346,046	754,780	1,809,163
NCig10036	10165-103B3	locus coeruleus, adult, donor10252	6,753,026	1,045,453	49,711	1,251,961	97,962	454,490	1,173,365	330,395	729,525	1,620,164
NCig10037	10166-103B4	cerebellum, adult, donor10252	6,025,035	1,095,992	54,415	368,370	62,650	519,173	650,125	254,418	492,016	2,527,876
NCig10038	11207-116A1	Endothelial Cells—Aortic, donor0	15,261,564	718,897	98,322	12,128,937	142,908	291,064	820,844	298,247	455,971	306,374
NCig10039	11222-116B7	Fibroblast—Gingival, donor4 (GFH2)	5,865,574	885,111	167,833	2,081,881	108,043	284,519	1,080,129	346,774	547,431	363,853
NCig10040	11224-116B9	CD14+ Monocytes, donor1	11,232,175	651,017	101,461	4,297,440	152,438	540,035	1,268,085	510,000	645,877	3,065,822
NCig10041	11229-116C5	CD14+ monocyte derived endothelial progenitor cells, donor1	10,775,321	1,032,309	242,145	3,950,479	190,791	539,087	1,666,613	561,795	835,546	1,756,556
NCig10042	11245-116E3	Fibroblast—Aortic Adventitial, donor1	9,543,436	735,498	828,670	3,376,827	198,604	517,858	2,135,913	655,705	710,317	384,044
NCig10043	11246-116E4	Intestinal epithelial cells (polarized), donor1	6,681,741	919,980	392,820	1,056,095	120,525	433,407	2,003,503	513,395	536,761	705,255
NCig10044	11247-116E5	Mesothelial Cells, donor1	7,150,721	870,202	443,481	1,547,197	127,726	418,103	2,291,855	516,801	516,012	419,344
NCig10045	11248-116E6	Anulus Pulposus Cell, donor1	9,329,478	673,123	467,283	4,733,836	191,255	418,230	1,674,689	474,236	571,373	125,453
NCig10046	11249-116E7	Pancreatic stromal cells, donor1	6,917,860	895,678	266,841	1,598,919	129,096	447,633	1,839,323	563,482	606,168	570,720
NCig10047	11256-116F5	Small Airway Epithelial Cells, donor1	8,934,394	762,215	197,286	3,113,793	175,723	506,591	2,330,196	629,638	801,987	416,965
NCig10048	11273-116H4	Mammary Epithelial Cell, donor1	10,019,381	890,533	198,834	4,561,811	208,742	497,865	2,025,351	497,139	721,321	417,785
NCig10049	11278-116H9	Placental Epithelial Cells, donor1	11,212,007	523,668	434,079	7,019,440	196,493	358,154	1,908,353	304,347	296,384	171,089
NCig10050	11282-116I4	Skeletal muscle cells differentiated into Myotubes—multinucleated, donor1	8,911,706	825,574	278,816	3,852,864	174,167	447,392	2,002,086	521,214	534,883	274,710
NCig10051	11468-119C1	Preadipocyte—omental, donor1	5,109,588	863,018	244,070	1,743,473	94,850	257,299	790,493	304,494	524,536	287,355
NCig10052	11487-119E2	Mast cell—stimulated, donor1	4,388,468	1,047,459	53,428	390,687	86,272	312,008	1,219,897	244,001	294,922	739,794
NCig10053	10411-106B6	renal cell carcinoma cell line:OS-RC-2	6,905,711	774,316	209,703	2,297,666	117,997	421,779	1,058,325	387,862	580,214	1,057,849
NCig10054	10412-106B7	malignant trichilemmal cyst cell line:DJM-1	9,858,285	728,347	139,130	3,031,554	164,712	630,434	2,045,672	648,552	895,267	1,574,617
NCig10055	10414-106B9	maxillary sinus tumor cell line:HSQ-89	9,125,063	857,517	135,611	2,250,778	123,989	579,817	1,951,209	498,578	697,019	2,030,545
NCig10056	10431-106D8	epidermoid carcinoma cell line:Ca Ski	5,074,986	1,071,422	146,593	982,637	87,543	343,508	859,004	378,966	452,570	752,743
NCig10057	10436-106E4	signet ring carcinoma cell line:Kato III	8,693,941	840,579	145,687	3,244,444	137,763	512,628	1,657,263	503,623	614,540	1,037,414
NCig10058	10442-106F1	schwannoma cell line:HS-PSS	7,714,618	941,029	176,159	1,799,562	134,668	519,866	1,659,980	589,180	733,263	1,160,911
NCig10059	10444-106F3	glioblastoma cell line:A172	8,266,061	861,701	175,094	2,804,921	186,736	495,954	1,209,931	520,670	712,755	1,298,299
NCig10060	10454-106G4	chronic myelogenous leukemia cell line:K562	4,756,581	1,045,797	109,272	645,627	70,593	363,272	675,740	342,295	400,380	1,103,605
NCig10061	10464-106H5	acute lymphoblastic leukemia (T-ALL) cell line:Jurkat	9,344,079	869,111	131,089	2,562,674	178,216	687,478	1,748,129	774,916	819,425	1,573,041
NCig10062	10508-107D4	neuroblastoma cell line:CHP-134, tech_rep1	4,622,691	962,974	148,947	278,618	57,421	405,741	662,738	258,098	391,938	1,456,216
NCig10063	10552-107I3	cervical cancer cell line:D98-AH2, tech_rep1	4,307,425	1,005,845	156,179	421,514	70,319	310,350	1,186,016	271,445	368,888	516,869
NCig10064	10558-107I9	osteosarcoma cell line:HS-Os-1, tech_rep1	4,374,077	983,856	182,894	548,737	80,879	357,116	711,651	286,493	395,130	827,321
NCig10065	10410-106B5	extraskeletal myxoid chondrosarcoma cell line:H-EMC-SS, tech_rep1	3,965,350	928,677	138,036	393,526	64,950	343,707	582,912	220,600	400,189	892,753
NCig10066	10441-106E9	synovial sarcoma cell line:HS-SY-II, tech_rep1	4,039,831	844,408	197,018	574,814	57,821	348,974	523,331	235,006	375,904	882,555
NCig10067	10474-106I6	myeloma cell line:PCM6, tech-rep1	4,582,185	810,459	186,856	755,594	71,301	358,371	807,453	278,280	416,507	897,364
NCig10068	10424-106D1	splenic lymphoma with villous lymphocytes cell line:SLVL	4,458,999	852,283	163,002	455,663	80,461	376,376	969,585	280,831	377,707	903,091
NCig10126	10508-107D4	neuroblastoma cell line:CHP-134, tech_rep2	5,259,146	995,795	48,625	550,450	63,938	396,327	701,319	298,112	438,554	1,766,026
NCig10127	10552-107I3	cervical cancer cell line:D98-AH2, tech_rep2	4,097,389	1,015,542	60,740	646,950	64,609	304,041	930,823	243,837	338,193	492,654
NCig10128	10558-107I9	osteosarcoma cell line:HS-Os-1, tech_rep2	4,681,628	968,282	75,235	865,891	76,828	336,463	737,523	296,891	409,283	915,232
NCig10129	10410-106B5	extraskeletal myxoid chondrosarcoma cell line:H-EMC-SS, tech_rep2	3,118,570	822,752	40,614	436,600	112,425	377,956	276,992	152,854	276,872	621,505
NCig10130	10441-106E9	synovial sarcoma cell line:HS-SY-II, tech_rep2	3,232,761	726,773	64,905	633,633	81,424	473,478	240,861	160,678	250,046	600,963
NCig10131	10474-106I6	myeloma cell line:PCM6, tech-rep2	3,985,344	720,124	60,988	898,043	78,483	322,369	566,406	231,781	346,042	761,108
The RNA identifier (Source.Name) can be searched in the FANTOM5 SSTAR database^15,20^. The RNA samples are also described in the SDRF files distributed alongside the FASTQ sequences and alignments, as well as the raw alignment statistics.												
